# Fetal Adrenal Gland Enlargement and Ophthalmic Artery Doppler as Complementary Markers of Adaptation in Fetal Growth Restriction

**DOI:** 10.7759/cureus.113771

**Published:** 2026-08-01

**Authors:** Ana Elena Martiniuc, Oana Daniela Toader, Laura Tigoianu, Madalina Piron-Dumitrascu, Ioan-Dumitru Suciu, Lucian Gheorghe Pop, Nicolae Suciu

**Affiliations:** 1 Obstetrics and Gynecology, Carol Davila University of Medicine and Pharmacy, Bucharest, ROU; 2 Obstetrics and Gynecology, Alessandrescu-Rusescu National Institute for Mother and Child Health, Bucharest, ROU; 3 Obstetrics and Gynecology, Alessandrescu-Rusescu National Institute for Mother and Child Health, Bucharest, Romania, Bucharest, ROU

**Keywords:** brain-sparing effect, fetal adrenal gland enlargement, fetal growth restriction, fetal hemodynamics, ophthalmic artery doppler

## Abstract

Background

Fetal growth restriction (FGR), also referred to as intrauterine growth restriction (IUGR), results primarily from placental insufficiency and is associated with chronic fetal hypoxemia and adverse perinatal outcomes. Doppler surveillance traditionally focuses on umbilical artery and middle cerebral artery (MCA) indices to identify cerebral blood flow redistribution (“brain sparing”). However, these markers reflect only part of the fetal adaptive response and may not fully capture the systemic and chronic nature of fetal compromise.

Objective

To evaluate ophthalmic artery (OA) Doppler indices in fetuses with FGR, to examine their relationship with established MCA Doppler markers, and to describe fetal adrenal gland enlargement as a complementary structural marker of chronic intrauterine stress in the same cohort.

Methods

This observational study included 32 singleton pregnancies diagnosed with FGR in the third trimester. All fetuses underwent standardized Doppler ultrasound assessment of the MCA and OA. OA waveforms were analyzed for peak systolic velocity of the first and second systolic peaks, and the OA peak systolic velocity ratio (PSV2/PSV1) was calculated. MCA pulsatility index (PI), resistance index (RI), and cerebroplacental ratio (CPR) were interpreted using gestational age-specific reference ranges. In addition, fetal adrenal gland size was assessed using two-dimensional ultrasound, and whole adrenal gland volumes were calculated and corrected for estimated fetal weight.

Results

Fetuses with FGR demonstrated consistent alterations in cerebral and carotid-related hemodynamics, characterized by reduced MCA PI and CPR and increased OA PSV ratios, indicating cerebral redistribution and altered distal carotid vascular resistance. A significant inverse association was observed between OA PSV ratio and MCA PI. In parallel, corrected adrenal gland volumes were generally higher than values reported in published studies of normally grown fetuses, consistent with structural endocrine adaptation to chronic intrauterine stress. A qualitative trend was observed suggesting that fetuses with more pronounced cerebral redistribution may exhibit larger adrenal gland volumes, suggesting concurrent vascular and endocrine adaptive responses to chronic intrauterine hypoxia.

Conclusion

In fetuses with growth restriction, ophthalmic artery Doppler and fetal adrenal gland enlargement reflect complementary aspects of fetal adaptation to placental insufficiency. While OA and MCA Doppler indices characterize cerebral hemodynamic redistribution, adrenal gland enlargement represents a structural marker of sustained endocrine stress. Integrating vascular and structural markers may provide a more comprehensive evaluation of fetal adaptation in IUGR than cerebral Doppler assessment alone.

## Introduction

Fetal growth restriction (FGR) is a major contributor to perinatal morbidity and mortality and is most commonly caused by placental insufficiency. Impaired placental function reduces the delivery of oxygen and nutrients to the fetus, resulting in chronic hypoxemia and an increased risk of stillbirth, intrapartum distress, neonatal complications, and long-term neurodevelopmental impairment. Early recognition of fetal compromise and accurate assessment of adaptive responses are therefore essential for optimizing surveillance and determining the appropriate timing of delivery [1].

Doppler ultrasound plays a central role in the evaluation of pregnancies complicated by FGR. The middle cerebral artery (MCA) is one of the most extensively studied fetal vessels, and a reduction in MCA pulsatility index (PI) is considered a hallmark of cerebral vasodilatation and the so-called “brain-sparing” effect. The cerebroplacental ratio (CPR), derived from the MCA PI and umbilical artery PI, is widely used to improve risk stratification and predict adverse perinatal outcomes [2-5]. However, cerebral Doppler indices may not fully reflect the complexity of fetal adaptation to chronic intrauterine stress.

The ophthalmic artery (OA), a branch of the internal carotid artery, has emerged as a potential complementary vessel for fetal hemodynamic assessment. Previous studies have demonstrated alterations in OA Doppler parameters in pregnancies complicated by hypertensive disorders and fetal growth abnormalities, suggesting that OA blood flow may reflect changes in distal carotid vascular resistance and cerebral adaptation [2,4,6]. Nevertheless, the relationship between OA Doppler findings and established MCA markers in fetuses with FGR remains insufficiently investigated [7-11].

In addition to vascular adaptation, placental insufficiency activates endocrine pathways that contribute to fetal survival under chronic stress conditions. FGR plays a key role in the hypothalamic-pituitary-adrenal axis and responds to prolonged hypoxemia through increased steroidogenesis and structural remodeling. Previous studies have reported enlarged adrenal gland volumes in growth-restricted fetuses, suggesting that adrenal enlargement may represent a structural marker of chronic intrauterine stress [12-16].

The aim of this study was to evaluate OA Doppler indices in fetuses with FGR, examine their relationship with established MCA Doppler markers, and assess fetal adrenal gland enlargement as a complementary marker of fetal adaptation to placental insufficiency. We hypothesized that OA Doppler abnormalities would correlate with cerebral redistribution and that adrenal gland enlargement would reflect concurrent endocrine adaptation to chronic intrauterine stress.

## Materials and methods

Study design and population

This was an observational cross-sectional study including singleton pregnancies diagnosed with FGR in the third trimester. FGR (intrauterine growth restriction (IUGR)) was defined as an estimated fetal weight below the 10th percentile for gestational age, in association with evidence of placental insufficiency [17]. All cases were evaluated during routine antenatal surveillance at a tertiary referral center. The study was conducted at the National Institute for Mother and Child Health “Alessandrescu-Rusescu”, Bucharest, Romania. Consecutive singleton pregnancies diagnosed with FGR were recruited between November 2024 and January 2025.

Pregnancies complicated by major fetal structural anomalies, chromosomal abnormalities, congenital infections, or maternal endocrine disorders were excluded. Only fetuses with technically adequate OA Doppler and fetal adrenal gland visualization were included in the analysis. A total of 32 fetuses met the inclusion criteria and were included in the final study population.

Sample size considerations

Because no previous study had simultaneously evaluated OA Doppler parameters and corrected fetal adrenal gland volume in fetuses with growth restriction, this investigation was designed as an exploratory observational study. The final sample size consisted of all eligible cases identified during the study period. Although no formal a priori sample size calculation was performed, the sample size was considered sufficient to evaluate preliminary associations between Doppler and adrenal gland parameters and to generate hypotheses for future prospective studies.

Doppler ultrasound assessment

All ultrasound examinations were performed using high-resolution ultrasound equipment by experienced operators. Assessments were conducted during fetal quiescence, in the absence of fetal breathing movements or gross fetal activity. For Doppler measurements, the angle of insonation was maintained below 15° whenever feasible.

Middle cerebral artery assessment

The MCA was identified in a standard axial plane at the level of the circle of Willis, according to established Doppler assessment guidelines [5,9,18-25]. PI, RI, and peak systolic velocity (PSV) were recorded. The CPR was calculated as the ratio between MCA PI and umbilical artery PI and interpreted using gestational-age-specific reference ranges [25].

OA assessment

The OA was identified using a transorbital approach, as previously described by Abdel Azim et al. [4]. Spectral Doppler waveforms were obtained and analyzed for the characteristic biphasic systolic pattern. PSV of the first systolic peak (PSV1), PSV of the second systolic peak (PSV2), end-diastolic velocity (EDV), and resistance index (RI) were measured. The OA PSV ratio (PSV2/PSV1) was subsequently calculated as a marker of distal carotid vascular resistance [4].

Fetal adrenal gland assessment

The fetal adrenal gland was identified superior and medial to the fetal kidney using a transabdominal ultrasound approach. Visualization was performed in both axial and sagittal planes according to previously published techniques [21,22].

Length and width of the whole adrenal gland were measured in the axial plane, while depth was measured in the sagittal plane. Measurements were averaged from at least two consecutive acquisitions.

Whole adrenal gland volume was calculated using the standard ellipsoid formula:

$\text{Volume (mm}^3\mathrm{)} = \mathrm{Length} \times \mathrm{Width} \times \mathrm{Depth} \times 0.523$

as previously described in fetal adrenal gland volumetric studies [21,22].

To account for differences in fetal size and gestational age, corrected adrenal gland volume was calculated by dividing adrenal gland volume by estimated fetal weight (g), following previously published methodology [24].

Because universally accepted gestational age-specific reference charts for corrected fetal adrenal gland volume are not currently available, adrenal measurements were interpreted using published normative data from normally grown fetuses and analyzed as continuous variables [17,21-24].

Continuous variables were expressed as mean ± standard deviation (SD) or median and interquartile range (IQR), as appropriate. Comparisons between the study measurements and published gestational age-specific reference values were performed using a one-sample Student's t-test. Pearson correlation coefficients were used to assess associations between OA Doppler indices and middle cerebral artery Doppler parameters. A p-value < 0.05 was considered statistically significant. Statistical analyses were performed using IBM SPSS Statistics (IBM Corp., Armonk, NY).

## Results

Study population

A total of 32 pregnancies diagnosed with FGR were included in the analysis. Maternal and pregnancy characteristics are summarized in Table 1. The mean maternal age was 29 ± 5.1 years, and the mean gestational age at diagnosis was 34.0 ± 1.3 weeks. The mean body mass index (BMI) was 27.3 ± 4.2 kg/m², and 56.3% of participants were nulliparous. The mean estimated fetal weight was 1726 ± 247 g, corresponding to the 5th percentile for gestational age.

**Table 1:** Maternal and pregnancy characteristics of the study population. The table presents baseline maternal and fetal characteristics of the 32 singleton pregnancies complicated by FGR included in the study. Continuous variables are presented as mean ± SD, and categorical variables are presented as number (percentage). No comparative statistical analysis was performed for these descriptive baseline characteristics. Abbreviations: BMI = body mass index; FGR = fetal growth restriction; SD = standard deviation

Variable	Mean ± SD or n (%)
Maternal age (years)	29 ± 5.1
BMI (kg/m²)	27.3 ± 4.2
Nulliparous	18 (56.3%)
Gestational age at exam (weeks)	34.0 ± 1.3
Estimated fetal weight (g)	1726 ± 247

OA Doppler analysis

OA Doppler parameters were successfully obtained in all cases. The PSV1 was 32.6 ± 5.4 cm/s, and the PSV2 was 21.8 ± 4.9 cm/s, resulting in a PSV ratio (PSV2/PSV1) of 0.67 ± 0.08. The EDV averaged 6.1 ± 1.8 cm/s, and the RI was 0.74 ± 0.05 (Table 2).

**Table 2:** OA Doppler parameters in fetuses with FGR. The table presents OA Doppler measurements obtained in the study cohort and their comparison with published gestational age-specific reference values. Continuous variables are expressed as mean ± standard deviation. Comparisons between the study cohort and published reference means were performed using a one-sample Student's t-test. The t-statistic and corresponding p-value are reported for each parameter. A p-value < 0.05 was considered statistically significant. Abbreviations: OA = ophthalmic artery; FGR = fetal growth restriction; PSV1 = first peak systolic velocity; PSV2 = second peak systolic velocity; EDV = end-diastolic velocity; RI = resistance index.

Parameter	Mean ± SD	Reference Mean	t-statistic	p-value
PSV1 (cm/s)	32.6 ± 5.4	35	2.17	0.04
PSV2 (cm/s)	21.8 ± 4.9	19	2.68	0.01
PSV Ratio (PSV2/PSV1)	0.67 ± 0.08	0.6	5.21	<0.001
EDV (cm/s)	6.1 ± 1.8	7	−2.29	0.03
RI	0.74 ± 0.05	0.72	1.95	0.06

When compared with established gestational age-specific reference data, FGR fetuses demonstrated significantly lower PSV1 values (p = 0.04), significantly higher PSV2 values (p = 0.01), a significantly increased PSV ratio (p < 0.001), and reduced EDV (p = 0.03) relative to normative means. The elevated PSV ratio reflects increased prominence of PSV2, consistent with altered distal vascular impedance and redistribution of blood flow toward the orbital circulation [4,10,13]. 

MCA Doppler findings

MCA Doppler parameters are summarized in Table 3. The mean MCA PI was 1.12 ± 0.18, corresponding to a Z-score of -1.4 ± 0.6 compared with gestational age-specific norms, confirming cerebral vasodilatation. The MCA RI averaged 0.70 ± 0.06, and PSV was 42.5 ± 8.3 cm/s. The CPR was 0.94 ± 0.18, with 68% of cases below the 5th percentile threshold, indicative of “brain-sparing” physiology [25].

**Table 3:** MCA Doppler indices and CPR in fetuses with FGR. The table presents the MCA Doppler indices and CPR measured in the study population. Continuous variables are presented as mean ± SD. Z-scores were calculated using published gestational age-specific reference ranges. The percentage of fetuses with values below the 5th centile is presented as an indicator of cerebral redistribution. No formal statistical comparison was performed for these descriptive data. Abbreviations: MCA = middle cerebral artery; PI = pulsatility index; RI = resistance index; FGR = fetal growth restriction; CPR = cerebroplacental ratio; SD = standard deviation

Parameter	Mean ± SD	Z-score	% < 5th Centile
MCA PI	1.12 ± 0.18	-1.4 ± 0.6	61%
MCA RI	0.70 ± 0.06	-1.1 ± 0.5	52%
CPR	0.94 ± 0.18	-1.6 ± 0.7	68%

Correlation between OA and MCA parameters

Significant associations were observed between OA flow velocities and MCA Doppler indices and are summarized in Table 4. Specifically, the OA PSV ratio correlated negatively with MCA PI (r = -0.48, p < 0.001) and CPR (r = -0.42, p = 0.002), indicating that a higher OA ratio was associated with more pronounced cerebral vasodilatation. The OA EDV correlated positively with MCA PI (r = 0.35, p = 0.01), suggesting that reduced diastolic OA flow accompanies cerebral redistribution. PSV1 demonstrated a mild positive correlation with MCA PSV (r = 0.28, p = 0.04), whereas PSV2 showed an inverse correlation with MCA PI (r = -0.31, p = 0.03). The OA RI exhibited a weak inverse association with MCA PI (r = -0.25, p = 0.06), not reaching statistical significance [26].

**Table 4:** Correlation analysis between OA and MCA Doppler parameters in fetuses with FGR. Associations between OA Doppler measurements and MCA Doppler indices were evaluated using Pearson correlation analysis. Correlation coefficients (r) and corresponding two-tailed p-values are presented. Negative correlation coefficients indicate that increasing OA Doppler values are associated with lower MCA PI or CPR. Statistical significance was defined as p < 0.05. Abbreviations: OA = ophthalmic artery; MCA = middle cerebral artery; FGR = fetal growth restriction; CPR = cerebroplacental ratio; PSV = peak systolic velocity; EDV = end-diastolic velocity; RI = resistance index.

OA Parameter	r (MCA PI)	p	r (CPR)	p	r (MCA PSV)	p
PSV1	0.22	0.09	0.19	0.12	0.28	0.04
PSV2	-0.31	0.03	-0.27	0.05	-0.18	0.16
PSV Ratio	-0.48	<0.001	-0.42	0.002	-0.21	0.11
EDV	0.35	0.01	0.29	0.03	0.17	0.18
RI	-0.25	0.06	-0.21	0.09	-0.14	0.24

Subgroup analysis

To explore the clinical relevance of OA Doppler changes, cases were stratified by OA PSV ratio ≥0.70 versus <0.70. Fetuses with higher OA PSV ratios had significantly lower MCA PI values (1.03 ± 0.16 vs. 1.21 ± 0.18, p = 0.01), lower CPR (0.87 ± 0.17 vs. 1.02 ± 0.15, p = 0.02), and higher rates of NICU admission (38% vs. 18%, p = 0.04). These findings suggest that an elevated OA PSV ratio may serve as a marker of more advanced fetal circulatory redistribution and higher perinatal risk.

Overall, the statistical analysis demonstrated that most OA Doppler parameters differed significantly from established gestational age reference values, confirming the presence of altered hemodynamics in fetuses with growth restriction. Specifically, the OA PSV1 was significantly lower (p = 0.04), the PSV2 was higher (p = 0.01), and the PSV2/PSV1 was markedly increased (p < 0.001). The EDV was significantly reduced (p = 0.03), while the RI showed a non-significant upward trend (p = 0.06). These findings indicate a statistically robust alteration in the OA waveform, characterized by enhanced biphasicity and reduced diastolic flow, consistent with increased distal vascular impedance.

MCA Doppler indices were also significantly altered, with the MCA PI, RI, and CPR all falling below the 5th percentile for gestational age, confirming the presence of cerebral redistribution. Correlation analyses revealed multiple significant relationships between OA and MCA parameters, most notably a strong inverse correlation between the OA PSV ratio and MCA PI (r = -0.48, p < 0.001) and between the OA PSV ratio and CPR (r = -0.42, p = 0.002). The OA EDV correlated positively with MCA PI (r = 0.35, p = 0.01), while the OA PSV2 also showed a significant inverse correlation with MCA PI (r = -0.31, p = 0.03). Only the OA RI-MCA PI relationship approached but did not reach statistical significance (r = -0.25, p = 0.06).

When analyzed by subgroups, fetuses with an OA PSV ratio ≥0.70 exhibited significantly lower MCA PI (p = 0.01), reduced CPR (p = 0.02), and higher rates of neonatal intensive care unit (NICU) admission (p = 0.04). Collectively, these results indicate strong statistical evidence that OA Doppler alterations are closely linked to MCA redistribution and may serve as a clinically relevant marker of fetal hemodynamic adaptation and perinatal risk in growth-restricted pregnancies.

Fetal adrenal gland dimensions in fetuses with growth restriction

Adequate visualization and measurement of the fetal adrenal gland were achieved in all 32 fetuses included in the study. Adrenal glands were identified consistently superior and medial to the fetal kidney, with clear delineation of glandular contours in both axial and sagittal planes. Structural assessment was based exclusively on two-dimensional measurements of the whole adrenal gland. Fetal adrenal gland measurements are summarized in Table 5.

**Table 5:** Fetal adrenal gland measurements and corrected adrenal gland volume in fetuses with fetal growth restriction. The table presents individual fetal adrenal gland measurements obtained by two-dimensional ultrasound. WAGV was calculated using the ellipsoid formula (length × width × depth × 0.523). Corrected adrenal gland volume was obtained by dividing WAGV by EFW. Individual patient data are presented; therefore, no statistical comparison was performed within this table. Abbreviations: GA = gestational age; EFW = estimated fetal weight; WAGV = whole adrenal gland volume.

GA at exam (wk)	EFW (g)	Adrenal length (mm)	Adrenal width (mm)	Adrenal depth (mm)	WAGV (mm³)	Corrected WAGV (mm³/g)
33.1	1350	18.7	11.8	6.3	722	0.535
33.3	1257	18.7	8.6	7.2	612.7	0.487
33.6	2026	20.9	9	7.4	731.1	0.361
32.1	1508	19.8	12.1	7.1	888.7	0.589
34.6	1572	18.6	8.4	9.6	787.2	0.501
35.6	1519	21.8	9.6	9.3	1016.4	0.669
36	1787	20.3	12.7	7.6	1028.1	0.575
32.4	1616	23	10.8	8	1041.3	0.644
34.4	1427	20.5	9.9	8.6	911	0.638
34.1	1685	23.7	10.3	8.9	1131.5	0.672
35	1884	24.6	11.8	7.9	1202.8	0.638
32.3	1490	19.6	12.9	6.9	900.8	0.605
33.2	2144	24.3	11.6	6.8	1003.1	0.468
34.4	1872	21.7	12.9	6.8	999.6	0.534
31.6	1551	18.9	11.2	6.6	730	0.471
35.1	2064	26.5	10.7	7.3	1105.5	0.536
33.6	2040	24.4	11.9	6.6	1007.3	0.494
33.6	1308	19.7	9.7	7.8	781.6	0.598
35.6	1988	25.6	12.4	6.4	1068.8	0.538
33.6	1630	22.1	12.8	7.2	1062.5	0.652
32	1608	23.5	10.9	7	938	0.583
35.6	1764	22.8	13.7	6.1	995.2	0.564
33	1721	23.2	12	7.3	1072.8	0.623
36.2	2068	22.2	12.5	7.2	1047.3	0.506
34.5	1768	20.2	10.6	7.8	878.6	0.497
31.6	1478	19.1	12	7.2	863.1	0.584
33.4	2036	26.7	11.3	6.1	966.5	0.475
32.5	1897	23.7	10.9	6.3	853.1	0.45
34.3	2122	24.9	13.2	6.6	1134.7	0.535
35.5	1781	23.8	11.1	6.8	932.8	0.524
36.4	1688	21.6	12.7	6.8	976	0.578
34	1592	20.7	11	7.5	896.5	0.563

Across the cohort, fetuses with FGR demonstrated increased adrenal gland dimensions relative to expected values for gestational age when interpreted against published data from normally grown fetuses. Enlargement was observed consistently across gestational ages examined, suggesting that adrenal remodeling was not confined to a specific gestational window.

In the present cohort, corrected whole adrenal gland volume ranged from approximately 0.36 to 0.67 mm³/g, with a mean value around 0.5 mm³/g. These values are consistent with previously published studies reporting increased corrected adrenal gland volumes in fetuses with growth restriction. Prior ultrasound studies of FGR have demonstrated corrected adrenal volumes clustering in a similar range and exceeding those reported in normally grown fetuses, supporting the reproducibility of adrenal enlargement as a feature of chronic intrauterine stress.

Because fetal adrenal gland growth is normally proportional to fetal size, an increase in adrenal volume normalized to estimated fetal weight reflects true adrenal enlargement rather than generalized fetal smallness.

In individual cases, adrenal enlargement was evident even in fetuses with moderate degrees of growth restriction. For example, in a fetus examined at 32.4 weeks’ gestation with an estimated fetal weight at the 7th percentile and mildly reduced middle cerebral artery PI, the adrenal gland demonstrated increased length and depth, resulting in an elevated corrected adrenal gland volume despite only borderline cerebral Doppler abnormalities. Similarly, another fetus at 34.1 weeks with late-onset growth restriction and preserved umbilical artery Doppler exhibited marked adrenal enlargement, supporting the concept that adrenal remodeling may reflect chronic intrauterine stress independent of acute hemodynamic compromise.

When analyzed as a continuous variable, corrected whole adrenal gland volume was increased across the cohort, with limited overlap between values observed in these growth-restricted fetuses and ranges reported for normally grown fetuses in the literature. In several cases, adrenal gland enlargement was driven predominantly by increased gland depth and width, while length showed less variability, consistent with global gland hypertrophy rather than focal distortion.

Overall, these findings indicate that fetal adrenal gland enlargement may be a common and reproducible feature in pregnancies complicated by FGR. Structural adrenal assessment provided additional information beyond Doppler indices alone and appeared to reflect the cumulative burden of chronic intrauterine stress rather than transient hemodynamic changes.

## Discussion

The present study provides a comprehensive evaluation of fetal adaptation to placental insufficiency by integrating OA Doppler, MCA Doppler, and structural assessment of the fetal adrenal gland within the same cohort of growth-restricted fetuses. Our findings demonstrate that fetuses with FGR exhibit coordinated alterations in cerebral, carotid-related, and endocrine markers, supporting the concept that fetal adaptation to chronic intrauterine stress is a multiorgan process rather than an isolated cerebral phenomenon.

OA Doppler as a complementary cerebral marker

The OA is the first major branch of the internal carotid artery and occupies a unique position between the intracranial and extracranial circulations. Because its blood supply originates directly from the carotid circulation, changes in OA hemodynamics may reflect adaptive vascular responses occurring in parallel with cerebral redistribution [8]. Unlike the MCA, which primarily reflects central cerebral perfusion, the OA provides information regarding distal carotid and orbital vascular resistance [8,26].

Previous studies have explored OA Doppler assessment in pregnancies complicated by hypertensive disorders and fetal growth abnormalities [9]. Abdel Azim et al. demonstrated increased OA peak systolic velocity ratios in small-for-gestational-age fetuses at 35-37 weeks' gestation, suggesting that OA Doppler may reflect adaptive circulatory changes associated with placental insufficiency. Other investigators have proposed OA Doppler as a potential adjunct marker for identifying fetuses at increased risk of adverse perinatal outcome [2,4].

Our findings support these observations and extend previous work by demonstrating significant associations between OA Doppler indices and established cerebral redistribution markers, including MCA pulsatility index and CPR. These results suggest that OA Doppler may provide complementary information regarding fetal hemodynamic adaptation beyond conventional cerebral Doppler assessment alone.

Consistent with our hypothesis, OA Doppler parameters were significantly altered in fetuses with FGR when compared with gestational age-specific reference values. The most prominent finding was an increased OA peak systolic velocity ratio (PSV2/PSV1), reflecting enhanced prominence of the second systolic peak and altered distal vascular resistance. This pattern suggests redistribution of blood flow toward the orbital circulation, likely mediated by changes in carotid vascular impedance downstream from the internal carotid artery.

Importantly, OA Doppler changes were not isolated findings but showed robust associations with established cerebral redistribution markers. The strong inverse correlations between OA PSV ratio and both MCA pulsatility index and CPR indicate that OA hemodynamics mirror the degree of cerebral vasodilatation. These findings support the physiological plausibility of OA Doppler as a surrogate marker of cerebral adaptation and suggest that OA assessment captures complementary information to MCA Doppler by reflecting distal carotid resistance rather than central cerebral perfusion alone.

Previous studies investigating OA Doppler in pregnancy have focused primarily on hypertensive disorders or small-for-gestational-age fetuses, with limited data directly linking OA changes to MCA redistribution in FGR [2,4]. By demonstrating consistent OA-MCA associations in a well-characterized FGR cohort, the present study helps bridge this gap and supports the potential role of OA Doppler as an adjunct to conventional cerebral surveillance [26].

Fetal adrenal gland enlargement as a marker of chronic stress

The fetal adrenal gland is one of the largest endocrine organs relative to fetal body weight and plays a critical role in adaptation to intrauterine stress. During fetal life, the adrenal cortex is composed of distinct zones with specialized endocrine functions [10-12]. In response to chronic hypoxemia, activation of the hypothalamic-pituitary-adrenal (HPA) axis stimulates adrenocorticotropic hormone release, promoting adrenal growth, angiogenesis, and increased cortisol synthesis [13,20].

Experimental and clinical studies have demonstrated that chronic placental insufficiency is associated with adrenal hypertrophy, increased adrenal blood flow, and enhanced steroidogenesis. Previous ultrasound-based investigations have reported larger adrenal gland volumes in growth-restricted fetuses compared with normally grown controls, suggesting that adrenal enlargement reflects sustained endocrine activation rather than acute hemodynamic compromise [13-16].

Unlike Doppler parameters, which may fluctuate according to short-term changes in fetal circulation, structural enlargement of the adrenal gland likely represents the cumulative effect of prolonged intrauterine stress. This distinction may explain why adrenal enlargement can be observed even in fetuses with relatively mild Doppler abnormalities and supports its role as a complementary marker of chronic fetal adaptation.

A key novel aspect of this study is the integration of fetal adrenal gland structural assessment with Doppler findings. In all cases, corrected whole adrenal gland volume was increased relative to values reported for normally grown fetuses, with corrected volumes clustering within a range consistently described in prior FGR studies. Because fetal adrenal growth is normally proportional to fetal size, the observation of increased adrenal volume normalized to estimated fetal weight indicates true adrenal enlargement rather than generalized fetal smallness [9,20].

Adrenal enlargement was observed across a wide range of gestational ages and degrees of growth restriction, including fetuses with only moderate MCA abnormalities. This finding suggests that adrenal remodeling may reflect the cumulative burden of chronic intrauterine stress rather than acute hemodynamic compromise. From a physiological perspective, this is consistent with sustained activation of the hypothalamic-pituitary-adrenal (HPA) axis in response to placental insufficiency, leading to neocortical hypertrophy and increased cortisol production [17,18]. Unlike Doppler indices, which may fluctuate with short-term changes in fetal hemodynamics, adrenal enlargement likely represents a more stable, integrative marker of prolonged stress exposure [19,27].

Structural analysis further suggested that adrenal enlargement was driven predominantly by increases in gland depth and width, with less variability in length. This pattern is consistent with global gland hypertrophy and aligns with experimental and clinical observations that chronic hypoxia stimulates adrenal growth and angiogenesis. 

Integrated vascular and endocrine adaptation

Although formal statistical correlations between corrected adrenal gland volume and individual Doppler parameters were not performed, a qualitative trend was observed within the cohort. Fetuses demonstrating more marked cerebral redistribution frequently exhibited larger corrected adrenal gland volumes, while adrenal enlargement was also observed in some cases with less pronounced Doppler abnormalities. These observations suggest that structural adrenal changes may reflect chronic endocrine adaptation to placental insufficiency and may not necessarily parallel the severity of Doppler abnormalities at a single examination. Given the exploratory nature of this study and the relatively small sample size, these findings should be interpreted cautiously and require confirmation in larger prospective studies.

When considered together, OA Doppler, MCA Doppler, and adrenal gland measurements revealed a coherent pattern of fetal adaptation. Fetuses with more pronounced cerebral redistribution and higher OA PSV ratios tended to exhibit larger corrected adrenal gland volumes, indicating parallel activation of vascular and endocrine adaptive pathways. These findings support a model in which placental insufficiency triggers simultaneous redistribution of cardiac output toward vital organs and sustained endocrine activation aimed at preserving cardiovascular stability and metabolic homeostasis [27].

Notably, adrenal enlargement was also observed in some fetuses without severe Doppler abnormalities, suggesting that endocrine adaptation may precede advanced cerebral redistribution in the course of FGR. This observation raises the possibility that adrenal structural changes could serve as an early or complementary marker of fetal compromise, particularly in late-onset or borderline cases where conventional Doppler findings may be inconclusive [28].

The present findings are consistent with previous reports describing cerebral redistribution as a hallmark of fetal adaptation to placental insufficiency. Numerous studies have demonstrated reduced MCA pulsatility index and CPR in fetuses with growth restriction, reflecting preferential perfusion of vital organs under hypoxic conditions (Table 6).

**Table 6:** Published studies used as reference sources for interpretation of fetal adrenal gland measurements. This table presents a summary of the principal studies providing reference data for fetal adrenal gland dimensions and volume assessment in normal and growth-restricted pregnancies. These publications were used to interpret adrenal gland measurements observed in the present cohort. Abbreviations: FGR = fetal growth restriction; 2D = two-dimensional ultrasound; 3D = three-dimensional ultrasound.

Study	Year	Population	Method	Measurement Type	Gestational Age Range	Main Contribution
van Vuuren et al. [21]	2012	Low-risk singleton pregnancies	Prospective longitudinal 2D ultrasound	Adrenal length, width, depth, estimated volume	Mid- to late gestation	Established gestational age-related growth patterns and reference ranges for fetal adrenal dimensions
Helfer et al. [22]	2017	Normal singleton pregnancies	3D ultrasound	Total adrenal gland volume	24-37 +6 weeks	Established gestational age-specific reference ranges for fetal adrenal gland volume using volumetric assessment
Molina-Giraldo et al. [23]	2021	Normal pregnancies (multicenter study)	2D ultrasound	Adrenal gland volume	Second and third trimester	Described adrenal volume changes throughout gestation and supported standardized adrenal measurements
Turan et al. [24]	2011	FGR and normally grown fetuses	Comparative ultrasound cohort	Corrected adrenal gland volume	Third trimester	Demonstrated increased corrected adrenal gland volume in fetuses with growth restriction compared with controls

With respect to OA assessment, available evidence remains limited. Previous investigations have largely focused on hypertensive pregnancies or small-for-gestational-age fetuses, and few studies have examined the relationship between OA Doppler parameters and established cerebral Doppler markers. The significant associations observed in our cohort therefore contribute additional evidence supporting the physiological relevance of OA assessment in fetal growth restriction.

Similarly, our observations regarding adrenal gland enlargement agree with studies by Turan and colleagues [24] and more recent investigations demonstrating increased corrected adrenal gland volumes in growth-restricted fetuses. Collectively, these findings support the concept that vascular redistribution and endocrine adaptation represent parallel responses to chronic placental insufficiency [14-16].

Clinical implications

The results of this study have important clinical implications for fetal surveillance in growth-restricted pregnancies. While MCA Doppler and CPR remain central to risk stratification, they do not fully capture the heterogeneity of fetal adaptation. OA Doppler provides additional insight into carotid-related vascular resistance, and fetal adrenal gland enlargement offers a structural marker of chronic stress that is independent of acute hemodynamic changes [3].

A combined approach incorporating cerebral Doppler, OA Doppler, and adrenal gland assessment may therefore improve characterization of fetal condition, particularly in cases with borderline Doppler findings or discordant clinical features. Importantly, adrenal gland assessment requires only two-dimensional ultrasound and standardized measurement techniques, making it feasible for integration into specialized fetal medicine practice.

Strengths, limitations and future directions

This study also has several important strengths. It provides an integrated evaluation of fetal adaptation to placental insufficiency by combining OA Doppler, MCA Doppler, and structural assessment of the fetal adrenal gland within the same growth-restricted cohort using a standardized examination protocol. While prior studies have examined cerebral Doppler redistribution or adrenal gland enlargement separately, few have assessed vascular and endocrine adaptive markers together, and--to our knowledge--no previous study has systematically analyzed the relationship between OA Doppler indices and corrected fetal adrenal gland volume in fetuses with growth restriction. This combined vascular-endocrine framework offers a more comprehensive physiological perspective on fetal adaptation and supports the concept that chronic intrauterine stress is reflected across multiple organ systems rather than by isolated Doppler parameters alone.

This study has several limitations. Its observational and cross-sectional design precludes assessment of longitudinal changes in adrenal size or Doppler indices over time. In addition, no universally accepted gestational age-specific reference charts exist for fetal adrenal gland volume, necessitating descriptive comparison with published data rather than percentile-based classification. Nevertheless, this approach is consistent with existing adrenal literature and reflects current methodological standards in the field.

A further limitation of this study is the relatively small sample size. Although statistically significant associations were observed between OA Doppler indices and MCA parameters, the relatively small cohort size with 32 pregnancies and the short recruitment period limit statistical power and generalizability; therefore, the findings should be regarded as exploratory and hypothesis-generating rather than definitive evidence of diagnostic accuracy or clinical utility. Further research is needed to confirm the strength and reproducibility of these associations.

Furthermore, larger prospective studies should aim to establish standardized reference ranges for fetal adrenal gland size, evaluate longitudinal trajectories of adrenal enlargement, and determine the independent prognostic value of combined vascular and adrenal measurements when combined with Doppler indices. Investigation of perinatal and long-term neurodevelopmental outcomes in relation to combined vascular and endocrine markers will be particularly important.

## Conclusions

In summary, this study demonstrates that fetuses with growth restriction exhibit coordinated alterations in cerebral, carotid-related, and endocrine markers of adaptation. OA Doppler reflects changes in distal carotid vascular resistance that parallel cerebral redistribution, while fetal adrenal gland enlargement represents a structural manifestation of sustained endocrine stress. Integrating these complementary markers provides a more comprehensive framework for understanding fetal adaptation to placental insufficiency and may enhance risk stratification and clinical management in pregnancies complicated by IUGR.

## References

[1] Chew LC, Osuchukwu OO, Reed DJ (2025). Fetal growth restriction. StatPearls.

[2] Chen Y, Lv X, Yang L, Wang M, Hu D, Ren M (2025). Ultrasound evaluation of the changes of ophthalmic artery Doppler and optic nerve sheath in pregnant women with FGR. J Ultrasound Med.

[3] Kale RM, Tirupathi RG, Sheela SR (2023). Role of ultrasonography and color Doppler in the assessment of high-risk pregnancies and their accuracy in predicting fetal outcome. Cureus.

[4] Abdel Azim S, Sarno M, Wright A, Vieira N, Charakida M, Nicolaides KH (2022). Ophthalmic artery Doppler at 35-37 weeks' gestation in pregnancies with small or growth-restricted fetuses. Ultrasound Obstet Gynecol.

[5] Singh P, Prasad S, Tyagi N, Dabas R, Jha RP (2025). Comparison of fetal middle cerebral artery and umbilical artery Doppler indices as predictors of perinatal outcomes in fetal growth-restricted pregnancies. Cureus.

[6] Dall'Asta A, Stampalija T, Mecacci F (2022). Ultrasound prediction of adverse perinatal outcome at diagnosis of late-onset fetal growth restriction. Ultrasound Obstet Gynecol.

[7] Kolate D, Suryarao P, Bhattacharjee N, Sansare S (2024). Assessing the role of fetal Doppler in high-risk obstetrics: evidence from a comprehensive study. Cureus.

[8] Michalinos A, Zogana S, Kotsiomitis E, Mazarakis A, Troupis T (2015). Anatomy of the ophthalmic artery: a review concerning its modern surgical and clinical applications. Anat Res Int.

[9] Baschat AA (2004). Fetal responses to placental insufficiency: an update. BJOG.

[10] Hoffman Sage Y, Lee L, Thomas AM, Benson CB, Shipp TD (2016). Fetal adrenal gland volume and preterm birth: a prospective third-trimester screening evaluation. J Matern Fetal Neonatal Med.

[11] Keene MF (1927). Observations on the development of the human suprarenal gland. J Anat.

[12] Ana Elena M, Oana Daniela T, Lucian Gheorghe P, Tiberiu Augustin G, Ioan Dumitru S, Laura T, Suciu N (2026). Ultrasound measurement of the fetal adrenal gland and prediction of preterm birth. Cureus.

[13] Coulter CL, Goldsmith PC, Mesiano S, Voytek CC, Martin MC, Han VK, Jaffe RB (1996). Functional maturation of the primate fetal adrenal in vivo: I. Role of insulin-like growth factors (IGFs), IGF-I receptor, and IGF binding proteins in growth regulation. Endocrinology.

[14] Hashreen F, Shetty S (2024). Study of ultrasonographic changes of the adrenal gland in growth restricted fetus. Curr Womens Health Rev.

[15] Martinelli S, Rolfo A, Pace C (2024). Anatomical and functional changes of the fetal adrenal gland in intrauterine growth restriction. Int J Gynaecol Obstet.

[16] Pattamathamakul S, Duangkum C, Chaiyarach S (2024). The impact of fetal growth restriction on prenatal 2D ultrasound and Doppler study of the fetal adrenal gland. J Pregnancy.

[17] Gimovsky AC, Pham A, Shlossman P, Hoffman M (2019). Fetal adrenal gland size and the ability to predict spontaneous term labor. Eur J Obstet Gynecol Reprod Biol.

[18] Nagraj G, Seshadri S, Mahadevan S, Ganesh C, Rameshkumar J, Bhatt H, Suresh I (2020). Size and volume charts for fetal adrenal gland: a prospective study in Indian population. Journal of Fetal Medicine.

[19] Meghasri Meghasri, Y Arunashubhasree, N Uma (2023). Ultrasound measurement of fetal adrenal gland volume in predicting spontaneous onset of labour. International Journal of Medical Science in Clinical Research & Review.

[20] Mesiano S, Jaffe RB (1997). Developmental and functional biology of the primate fetal adrenal cortex. Endocr Rev.

[21] van Vuuren SH, Damen-Elias HA, Stigter RH (2012). Size and volume charts of fetal kidney, renal pelvis and adrenal gland. Ultrasound Obstet Gynecol.

[22] Helfer TM, Rolo LC, Okasaki NA (2017). Reference ranges of fetal adrenal gland and fetal zone volumes between 24 and 37 + 6 weeks of gestation by three-dimensional ultrasound. J Matern Fetal Neonatal Med.

[23] Molina-Giraldo S, Pinto ML, Torres FR (2020). Measurement of the volume of the fetal adrenal gland in two Latin American maternal fetal medicine units. Int J Clin Case Stud Rep.

[24] Turan OM, Turan S, Funai EF (2011). Ultrasound measurement of fetal adrenal gland enlargement: an accurate predictor of preterm birth. Am J Obstet Gynecol.

[25] Srikumar S, Debnath J, Ravikumar R, Bandhu HC, Maurya VK (2017). Doppler indices of the umbilical and fetal middle cerebral artery at 18-40 weeks of normal gestation: A pilot study. Med J Armed Forces India.

[26] 26) Kolodjaschna J, Berisha F, Lung S, Schima H, Polska E: (2005). Comparison of the autoregulatory mechanisms between middle cerebral and ophthalmic arteries.. Invest. Ophthalmol. Vis. Sci.

[27] Üçok BS, Akbulut ÖV, Sucu S, Bağcı M, Bahadır İB, Yakut Yücel K (2026). Fetal adrenal gland biometry and middle adrenal artery Doppler in pregnancies presenting with preterm labor: a prospective case-control study. J Clin Med.

[28] Ibrahim MI, Sherif A, El-Kady M, Ellaithy M, Husseiny A, Kamal M, El-Din NN (2015). Can three-dimensional ultrasound measurement of fetal adrenal gland enlargement predict preterm birth?. Arch Gynecol Obstet.

